# Gender Differences in Fundamental Motor Skills Proficiency in Children Aged 3–6 Years: A Systematic Review and Meta-Analysis

**DOI:** 10.3390/ijerph19148318

**Published:** 2022-07-07

**Authors:** Yunfei Zheng, Weibing Ye, Mallikarjuna Korivi, Yubo Liu, Feng Hong

**Affiliations:** 1 College of Physical Education and Health Sciences, Zhejiang Normal University, Jinhua 321004, China; zyf123@zjnu.edu.cn; 2 Institute of Human Movement and Sports Engineering, Zhejiang Normal University, Jinhua 321004, China; ywbls@zjnu.cn (W.Y.); mallik.k5@gmail.com (M.K.); 3 Department of Sports Operation and Management, Jinhua Polytechnic, Jinhua 321007, China

**Keywords:** motor skills, child, Test of Gross Motor Development, sex differences

## Abstract

The age range of 3–6 years is considered as a critical period in developing and learning fundamental motor skills (FMS). To make the formulation of future FMS guidance programs more targeted, we examined gender differences in children’s FMS proficiency using a meta-analysis. Structured electronic databases including PubMed, Scopus and Web of Science were systematically searched using key terms, and the Joanna Briggs Institute (JBI) was used to assess the quality of included literature. Finally, 38 articles (39 studies) met the pre-specified inclusion criteria. The results showed that boys had higher proficiency in total FMS and object control skills than girls (SMD = 0.17 (95% CI 0.03, 0.31), *p* = 0.02; SMD = 0.48 (95% CI 0.38, 0.58), *p* < 0.00001), and gender differences in locomotor skill proficiency approached significance, trending in favor of girls (SMD = −0.07 (95 % CI −0.15, 0.01), *p* = 0.09, I^2^ = 66%). Meta-regression shows that age is associated with gender differences in object control skills (*p* < 0.05). In addition, through subgroup analysis, we found that boys’ advantage in object control skills increased with age (3 years: SMD = 0.27 (95% CI 0.00, 0.54), *p* < 0.00001; 4 years: SMD = 0.58 (95% CI 0.38, 0.77), *p* < 0.00001; 5 years: SMD = 0.59 (95% CI 0.31, 0.88), *p* < 0.00001; 6 years: SMD = 0.81 (95% CI 0.61, 1.01), *p* < 0.00001). In this meta-analysis, we found gender differences in FMS levels in children aged 3–6 years. Notably, gender differences in skill proficiency in object control were influenced by age. We recommend focusing on and developing girls’ object control skills starting at age 3.

## 1. Introduction

Regular participation in physical activity (PA) has potential benefits for children to improve obesity [1,2], bone health [3], psychological health [4] and cognitive function [5,6]. However, children’s physical activity levels worldwide are not positive. A study comparing physical activity behaviors of children from 15 countries found that PA behavioral indicator scores were generally low [7]. Studies have found a positive correlation between children’s fundamental motor skills (FMS) and PA, and FMS have been identified as a potential mechanism for the development of PA [8,9,10,11]. FMS refer to the basic abilities and skills for children to perform a series of organized basic movements, and they includes locomotor skills (e.g., running, jumping, sliding, etc.) and object control skills (e.g., hitting, catching ball, kicking, etc.) [12]. FMS play a vital role in using more professional and complex skills in playing, games and sports [13,14]. The learning and mastery of FMS play an important role in the healthy development of children. A previous study concluded that FMS in children were significantly associated with health-related fitness (HRF) components (body composition, muscular strength, muscular endurance, cardiovascular endurance) and that the effect increases with age [15]. Despite the many benefits of FMS, a recent systematic review showed that there is still much room for improvement in FMS globally in children of all ages within the range of 3–10 years [16].

It has been well established that FMS are crucial to a child’s development. When children are provided with few or no opportunities to achieve appropriate FMS levels, they are at risk of suffering from slowed motor development, thus limiting their chances for successful participation in an active and healthy sports culture [8]. Given the above, it seems crucial to improve children’s FMS. A recently published study protocol presents detailed experimental designs to investigate the effects of different physical activity interventions on FMS in children [17]. However, to meet the physical developmental needs of children, exercise programs should also be tailored to their unique developmental needs, so it is important to understand gender characteristics in FMS.

However, no unified conclusion has been reached on whether there are gender differences in FMS proficiency in children. Several studies have found gender differences in FMS in children [16,18,19,20], with boys having higher proficiency in object control skills than girls [16,18,19,21,22], In contrast, boys and girls have been found to have similar locomotor skills proficiency [18,19,21]. Pieces of evidence suggest gender differences in locomotor skills proficiency in children, with girls showing higher proficiency [20,22]. These inconsistent results may be clarified via a meta-analysis. To the best of our knowledge, no study has investigated age variation points for gender differences in motor skills. Hence, the main aim of the present study was to systematically review and provide a meta-analysis of the gender differences in FMS, locomotor skills and object control skills in children aged 3–6 years. Secondarily, this study aims to investigate the age pattern of gender differences in motor skill proficiency by meta-regression analysis and determine the age inflection points at which gender differences emerge.

## 2. Materials and Methods

The systematic review was performed in accordance with the Preferred Reporting Items for Systematic Reviews and Meta-Analyses (PRISMA) guidelines [23], and the study was registered with PROSPERO (CRD42021281160).

### 2.1. Eligibility Criteria

Articles assessing proficiency in FMS, locomotor skills and object control skills in children aged 3 to 6 years were included in this systematic review and meta-analysis. Studies were selected if they met the following criteria: (1) children aged 3–6 years; (2) scores reported by age and gender, if the study is an intervention study, with a baseline data report required; (3) results assessed using the Test of Gross Motor Development scale (TGMD), including modified versions; and (4) outcome measures reported using raw scores.

We excluded studies that met any of the following criteria: (1) review articles, conference abstracts or books; (2) participants not being assessed simultaneously according to age and gender; (3) inclusion of special populations such as those with disabilities or diseases; and (4) studies not published in English. We calculated the pooled effect size through meta-analysis.

Inclusion and exclusion criteria were formulated and applied by two reviewers (Y.Z. and M.K.) independently, and in the case of disagreement, they were confirmed by another reviewer (W.Y.).

### 2.2. Search Strategy

The literature search was conducted until 10 July 2021 by two independent reviewers (Y.Z. and Y.L.) with the following electronic databases: PubMed, Scopus and Web of Science (Supplementary Materials). Search terms included “child” OR “children” OR “preschoolers” OR “boy” OR “girl” AND “fundamental movement skills” OR “motor skills” OR “motor development” OR “gross motor” AND “TGMD” OR “Test of Gross Motor Development”. Specific search strategies can be found in the Supplementary Materials. In addition, we manually searched reference lists in some key previously published studies and reviews.

### 2.3. Data Extraction and Quality Assessment

Data were independently extracted by two reviewers (W.Y. and F.H.) and cross-checked, and controversial issues were discussed based on the original text to determine the final outcome. The extracted information included study characteristics (author name, publication time, country, study type), participant characteristics (environment, age, gender, sample), measurement information (outcome measures, outcome indicators, outcome), etc.

The two reviewers (M.K. and F.H.) independently assessed the quality of each included article using the Joanna Briggs Institute (JBI) quality appraisal checklists for cross-sectional, case–control, cohort studies, quasi-experimental and randomized control trials [24] (Supplementary Materials). If there were disagreements, we discussed these together until consensus was reached. The critical evaluation checklist for various research methods has 8 to 13 items, and positive responses were rated as “yes”. We identified studies with an overall positive response between 50% and 75% as studies with moderate quality, and studies with a positive response over 75% were considered to be of high quality.

### 2.4. Synthesis Methods

In this study, differences in FMS proficiency among boys and girls were compared using Review Manager version 5.4.1 from the Cochrane Assistance Network. The data in this paper are continuous variables, and the comprehensive effect index is the Standard Mean Difference (SMD) and its 95% CI. SMD was interpreted as very small (<0.2), small (0.2–0.5), moderate (0.5–0.8) and large (>0.8) [25], where *p* < 0.05 indicates significant differences between the genders. The I-squared (I^2^) statistic was used to test the heterogeneity between studies. When I^2^ ≤ 50, there was no heterogeneity between studies, and a fixed-effects model was used for meta-analysis; when I^2^ > 50, there was heterogeneity between studies, and a random-effects model was used for meta-analysis [26]. The pooled results showed heterogeneity between studies, which we addressed by meta-regression analysis and subgroup analysis. In addition, we sequentially excluded literature for sensitivity analysis, evaluated the stability of the combined results of the meta-analysis and verified the existence of publication bias in the included studies using Egger’s test.

When more than two subgroups needed to be merged in the research, the first two subgroups were merged first, then the third subgroup was merged, and so on. The merge formula is as follows [27]:

$$SD=\sqrt{\frac{(N_{1}-1)SD_{1}^{2}+\left(N_{2}-1\right)SD_{2}^{2}+\frac{N_{1}N_{2}}{\left(N_{1}+N_{2}\right)}\left(M_{1}^{2}+M_{2}^{2}-2M_{1}M_{2}\right)}{N_{1}+N_{2}-1}}$$

where *SD* is standard deviation; Group 1 sample size is *N*_1_, mean is *M*_1_, standard deviation is *SD*_1_; and Group 2 sample size is *N*_2_, mean is *M*_2_, and standard deviation is *SD*_2_.

## 3. Results

### 3.1. Search Results

**Figure 1:**
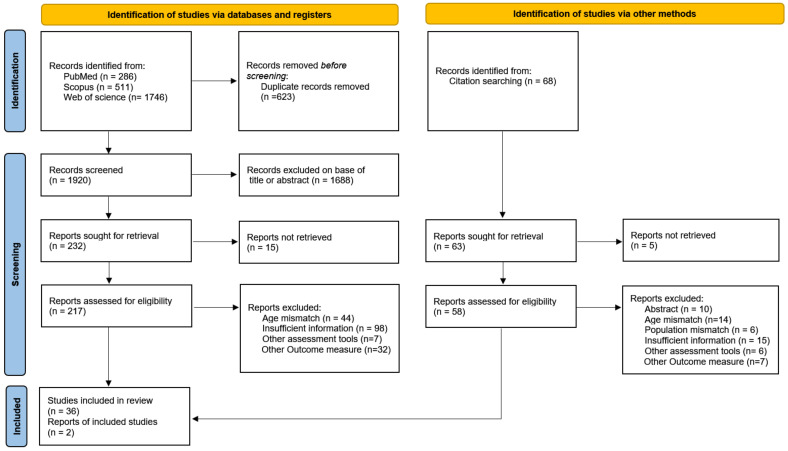
Flowchart of the study selection according to the Preferred Reporting Items for the Systematic Reviews and Meta-Analysis (PRISMA 2020) method.

A total of 2543 articles were retrieved through PubMed, Scopus and Web of Science databases, and 68 articles were obtained from reference lists of previously published studies and reviews. In the beginning, 623 duplicate records were removed from the 2543 articles. Of the remaining 1920 studies, 1688 irrelevant articles were excluded by reading the titles and abstracts, resulting in 232 items. After 15 articles that were not found were excluded, 217 articles were finally reviewed by full-text reading. From this stage, 44 articles were excluded due to age mismatch, 98 articles were removed because of insufficient information, 7 articles were excluded as they used other assessment tools, and 32 records were excluded due to other outcome measures. Ultimately, 36 articles met the inclusion criteria. In addition, 68 articles retrieved from the reference list were screened layer by layer, and 2 articles were finally included for the analysis. The detailed flow chart of the literature search, screening and selection is shown in Figure 1.

### 3.2. Characteristics of Included Studies and Quality Assessment

After the inclusion and exclusion criteria had been applied, a total of 38 articles (39 studies) were included in the systematic review and meta-analysis. These studies were carried out in 19 different countries as follows: Australia [28], Belgium [29], Brazil [30,31,32,33,34], Britain [35,36,37], China [38,39,40,41,42,43], Croatia [44], Germany [45], Iran [46], Indonesia [47,48], Ireland [49,50], Japan [51], Korea [52], Myanmar [53], Poland [54,55], Portugal [56,57], Puerto Rico [58], Singapore [59], South Africa [60,61] and the USA [29,62,63,64,65]. A total of 2598 children participated in the FMS assessment, 8837 children participated in the locomotor skills assessment, and 8394 children participated in the object control skills assessment, ranging in age from 3 to 6 years old. A total of 33 studies were cross-sectional studies [28,29,30,31,32,33,34,35,36,37,38,40,43,44,45,47,48,49,50,51,52,53,54,55,56,57,58,59,60,61,62,63,65], one was a case–control study [39], one was a cohort study [42], two were quasi-experimental studies [46,64], and one was a randomized controlled study [41]. There were 2 studies that used TGMD-1 [44,58], 30 that used TGMD-2 [28,29,30,31,32,35,36,37,38,39,40,41,43,46,47,50,51,52,53,54,55,56,57,59,60,61,62,63,64], 1 that used the modified version of TGMD-2 [33], 5 that used TGMD-3 [42,45,48,49,65], and 1 that used the modified version of TGMD-3 [34]. Based on the JBI quality evaluation criteria, the quality scores of the included studies ranged from 60% to 100%. Most of the included articles are of high quality, a few are of medium quality, and none of them are of low quality. Test locations, test items, score reports and quality assessments are shown in Table 1.

### 3.3. Gender Difference in Total FMS

**Figure 2:**
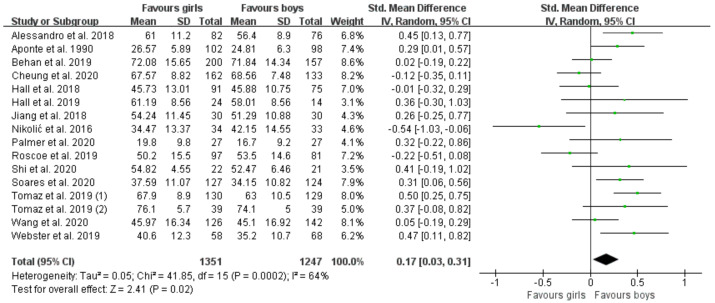
Forest plot of total FMS scores [30,32,35,36,37,39,40,41,42,44,49,58,60,61,64,65].

Sixteen studies assessed total FMS [30,32,35,36,37,39,40,41,42,44,49,58,60,61,64,65], including 1351 boys and 1247 girls. Figure 2 displays the forest plots of standardized mean differences and 95% CI for the total FMS score (16 studies) based on the random effects meta-analysis results. Significant differences favor boys vs. girls (SMD = 0.17 (95% CI 0.03, 0.31), *p* = 0.02, I^2^ = 64).

### 3.4. Gender Difference in Locomotor Skills

**Figure 3:**
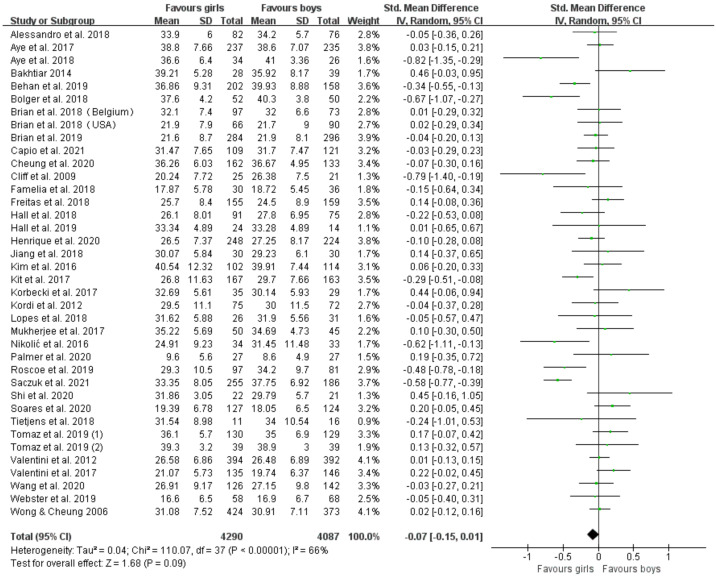
Forest plot of locomotor skills scores [28,29,30,31,32,33,34,35,36,37,38,39,40,41,42,43,44,45,46,47,48,49,50,51,52,53,54,55,56,57,59,60,61,62,63,64,65].

Thirty-seven articles (thirty-eight studies) assessed proficiency in locomotor skills [28,29,30,31,32,33,34,35,36,37,38,39,40,41,42,43,44,45,46,47,48,49,50,51,52,53,54,55,56,57,59,60,61,62,63,64,65], including 4290 boys and 4087 girls. Figure 3 displays the forest plots of standardized mean differences and 95% CI for the locomotor skills score (38 studies) based on the random effects meta-analysis results. Gender differences in locomotor skill proficiency approached significance, trending in favor of girls (SMD = −0.07 (95 % CI −0.15, 0.01), *p* = 0.09, I^2^ = 66%).

### 3.5. Gender Difference in Object Control Skills

**Figure 4:**
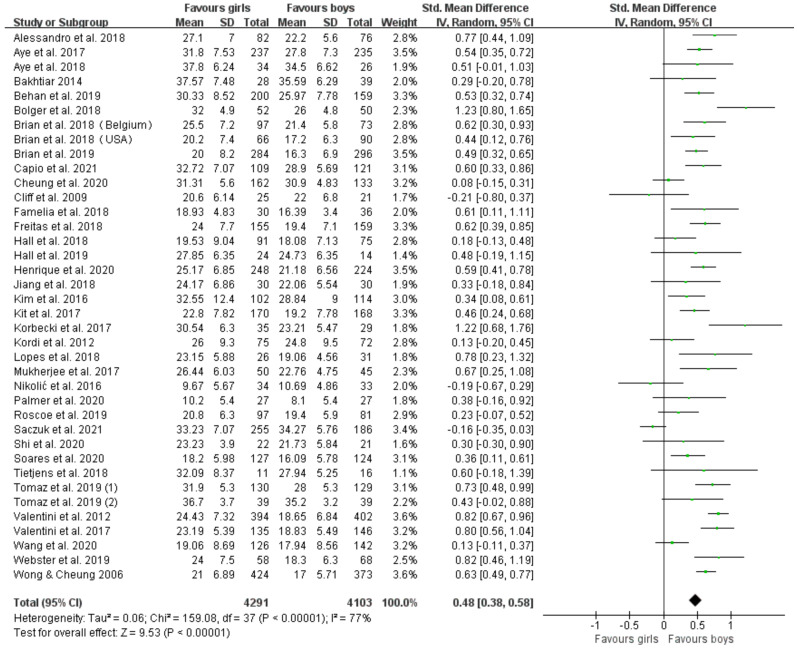
Forest plot of object control skills scores [28,29,30,31,32,33,34,35,36,37,38,39,40,41,42,43,44,45,46,47,48,49,50,51,52,53,54,55,56,57,59,60,61,62,63,64,65].

**Figure 5:**
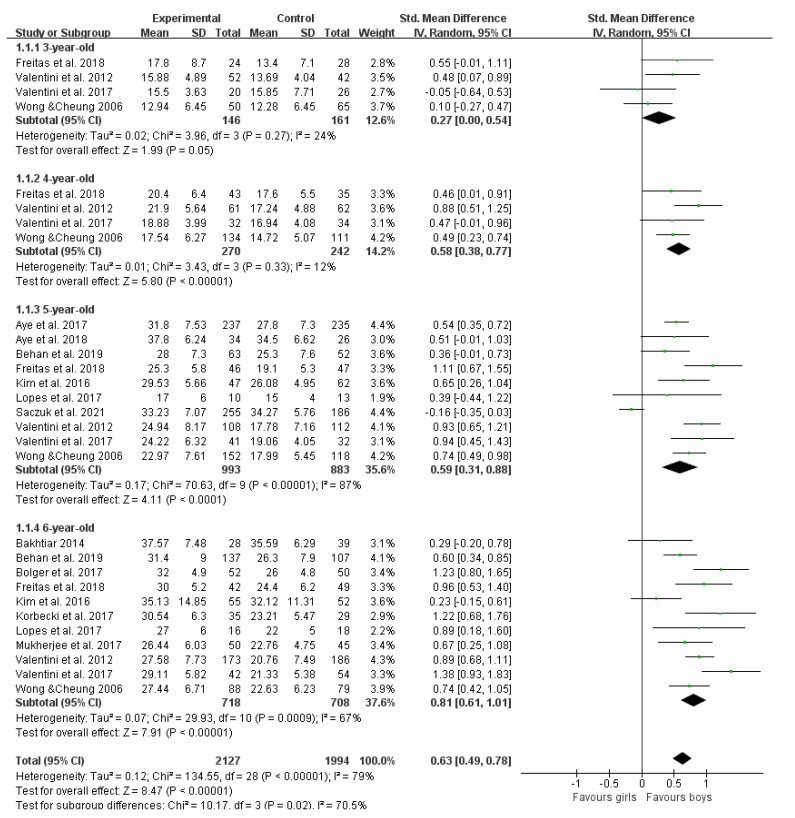
Forest plot of object control skills (age subgroups) [28,29,30,31,32,33,34,35,36,37,38,39,40,41,42,43,44,45,46,47,48,49,50,51,52,53,54,55,56,57,59,60,61,62,63,64,65].

Thirty-seven articles (thirty-eight studies) assessed proficiency in object control skills [28,29,30,31,32,33,34,35,36,37,38,39,40,41,42,43,44,45,46,47,48,49,50,51,52,53,54,55,56,57,59,60,61,62,63,64,65], including 4291 boys and 4103 girls. Figure 4 displays forest plots of the standardized mean differences and 95% CI for the object control skills score (38 studies) based on the random effects meta-analysis results. Significant differences were found, favoring boys vs. girls (SMD = 0.48 (95% CI 0.38, 0.58), *p* < 0.00001). Meta-regression displays that age is associated with gender differences in object control skills (*p* < 0.05). To further explore the effect of age, we divided studies with age-specific assessments into a 3 year-old group, a 4 year-old group, a 5 year-old group and a 6 year-old group. In subgroup analyses (Figure 5), we found marginally significant results favoring boys vs. girls in children aged 3 (SMD = 0.27 (95% CI 0.00, 0.54), *p* = 0.05) and significant results favoring boys vs. girls aged 4, 5 and 6 years (SMD = 0.58 (95% CI 0.38, 0.77), *p* < 0.00001; SMD = 0.59 (95% CI 0.31, 0.88), *p* < 0.00001; SMD = 0.81 (95% CI 0.61, 1.01), *p* < 0.00001), which increased with age.

### 3.6. Sensitivity Analysis and Publication Bias

After excluding 39 studies one by one, it was found that there was no significant change in the magnitude or direction of differences in the proficiency of children of different genders in terms of FMS, locomotor skills or object control skills.

Egger’s test was used to assess publication bias in FMS, locomotor skills and object control skills. The results showed that none of the studies included in the above review had publication bias (*p* > 0.05), as shown in Table 2.

## 4. Discussion

This systematic review and meta-analysis aggregated studies from Asia (China [38,39,40,41,42,43], Iran [46], Indonesia [47,48], Korean [52], Myanmar [53], Japan [51] and Singapore [59]), Africa (South Africa [60,61]), Europe (Belgium [29], Britain [35,36,37], Croatia [44], Germany [45], Ireland [49,50], Poland [54,55] and Portugal [56,57]), North America (the United States (US) [29,62,63,64,65] and Puerto Rico), Oceania (Australia [28]) and South America (Brazil [30,31,32,33,34]) and demonstrated gender differences in FMS proficiency in children aged 3–6 years. Combined results show that boys are more proficient than girls in total FMS proficiency. From the two dimensions of proficiency in locomotor skills and in object control skills, marginally significant differences were found favoring girls, and significant differences were found favoring boys in object control skills.

Differences in proficiency in object control skills between boys and girls seem to take some cues from biology. A study reported that boys are more likely to use finely segmented pelvic–torso–shoulder rotation when throwing [66]. Young explained the differences in human throwing and hitting behavior from an evolutionary perspective. Early humans made a living by throwing stones and swinging clubs. Women invested more resources into reproduction, and men were more likely to be hunters and warriors. These kinds of patterns are inherited through natural selection [67]. A previous study speculates that mature throwing is more likely an innate skill whose development is biologically determined and somewhat difficult to be influenced by nurture, and the same may be true of striking [68]. Sociological factors and behavior habits may also contribute to gender differences in proficiency in object control. Physical education programs are important for the development of FMS in preschoolers. Research shows that structured physical activity lessons can improve children’s FMS [69]. A meta-analysis shows that three or more teacher-led physical activity sessions per week significantly improved FMS [11]. Furthermore, studies have shown a correlation between FMS proficiency and physical activity levels in children. A study using TGMD-2 and accelerometers measured data on FMS and physical activity in kindergarteners and found a positive relationship between object control skills and moderate-intensity physical activity (MVPA) [70]. However, it has been shown that girls are significantly less likely to participate in physical activity than boys during the preschool years, especially at moderate to high intensity [71]. A systematic review including 10,316 children aged 3–6 years (5236 boys and 5080 girls) demonstrated that boys were more physically active than girls [72]. A survey in Norway showed that among children aged 3–4, only 32% of girls and 67% of boys were able to achieve the recommended 60 min of moderate to vigorous physical activity per day [73]. Therefore, different levels of physical activity may be responsible for the gender differences in object control scores. In addition, differences in exercise content may also contribute to gender differences in object control. A cross-sectional study from Japan showed that 5 year-old boys had significantly higher raw scores in terms of object control than girls of the same age (37.8 ± 6.24 vs. 34.5 ± 6.62, respectively), which is consistent with our findings, and the difference is mainly reflected in hitting, kicking and throwing [51]. A study in Australia showed that girls opted for dance and aerobic exercises far more often than boys [74]. A study found that Taiwanese girls prefer to play hopscotch, balance beam and house, while boys prefer ball games and slapstick games [75]. Previous studies have indicated that girls tend to lack opportunities to practice ball games, while boys generally spend more time participating in these games [76,77], which may also be related to parental educational attitudes [78]. In addition, an interesting study in Canada showed that 5 year-old girls’ perception of physical ability was related to their proficiency in locomotor skills, but not to object control skills, which may be because girls do not value object control skills [79].

Object control skills are more important than locomotor skills in childhood and continue to affect adolescence [44]. Evidence shows that gender disparities are reduced if girls have the same opportunities for mentoring, feedback, practice and encouragement [64]. Our meta-regression analysis revealed that age was the main factor influencing differences in proficiency in object control skills between boys and girls. Using subgroup analysis to further explore the effect of age, we found that gender differences in children’s proficiency in object control skill tend to be significant at age 3, and the advantage tends to favor boys. The difference is significant at the age of 4, and the advantage of boys begins to gradually increase with age, reaching a maximum at the age of 6. We recommend that parents and teachers should start paying attention to children’s movements when they are 3 years old and consciously guide children’s sports participation types; in particular, girls are encouraged to participate in ball games. Scholars should comprehensively consider the growth and development patterns, types of exercise and professional guidance of boys and girls when studying FMS guidance plans for children.

This meta-analysis provides evidence for gender differences in FMS proficiency in children aged 3–6 years, but some limitations should be considered. First, there are fewer articles and a smaller sample size for children aged 3 and 4 years, which requires more data to confirm. Second, our study only included children aged 3–6 years, and gender differences in FMS in children of other ages are also an important topic. Third, due to the limited number of articles, the study could not be specific to each item in the TGMD subscale (e.g., running, jumping, dribbling, etc.). Studies on specific TGMD items will therefore also be an interesting and useful topic as the number of high-quality studies increases. Finally, because TGMD is the most common tool for measuring FMS proficiency in educational, clinical and research settings, our study only included articles using TGMD or any modified version (TGMD-2 or TGMD-3), but this may have led to inconsistent results. Currently, assessments of children’s FMS competencies are primarily conducted through process-oriented and product-oriented approaches. TGMD is a process-oriented assessment that examines children’s motor performance on locomotor and object control tasks. We suggest that future research use product-oriented tools to further explore the gendered characteristics of FMS proficiency in young children.

## 5. Conclusions

Our findings demonstrated that there were gender differences in total FMS proficiency in children aged 3–6, with boys being more proficient than girls, and locomotor proficiency differences between gender approached significance, with a trend favoring girls. In the performance of proficiency in object control skills, boys were better than girls, and this difference gradually increased with age. We recommend focusing on and developing girls’ object control skills starting at age 3.

## Figures and Tables

**Table 1:** Characteristics of the included studies.

Author	Country	Design	Sample		Scale	Test Items	Outcome	Assessment
			Age	N (Boy/Girl)				
Alessandro et al. 2018 [30]	Brazil	CS	5–6	158 (82/76)	TGMD-2	LM, OC, FMS	FMS: ← LM: ↔ OC: ←	7
Aponte et al. 1990 [58]	Puerto Rico	CS	5–6	200 (102/98)	TGMD-1	FMS	FMS: ←	5
Aye et al. 2017 [53]	Myanmar	CS	5	472 (237/235)	TGMD-2	LM, OC	LM: ↔ OC: ←	7
Aye et al. 2018 [51]	Japan	CS	5	60 (34/26)	TGMD-2	LM, OC	LM: → OC: ←	7
Bakhtiar 2014 [47]	Indonesia	CS	6	67 (28/39)	TGMD-2	LM, OC	LM: ↔ OC: ↔	6
Behan et al. 2019 [49]	Ireland	CS	5–6	FMS: 357 (200/157) LM: 360 (202/158) OC: 359 (200/159)	TGMD-3	LM, OC, FMS	FMS: ↔ LM: → OC: ←	6
Bolger et al. 2017 [50]	Ireland	CS	6	102 (52/50)	TGMD-2	LM, OC	LM: → OC: ←	7
Brian et al. 2018 [29]	Belgium/USA	CS	4–5	Belgium: 170 (97/73) USA:156 (66/90)	TGMD-2	LM, OC	Belgium: LM: ↔ OC: ←USA: LM: ↔ OC: ←	6
Brian et al. 2019 [62]	USA	CS	3–6	580 (284/296)	TGMD-2	LM, OC	LM: ↔ OC: ←	7
Capio et al. 2021 [38]	China	CS	4–6	230 (109/121)	TGMD-2	LM, OC	LM: ↔ OC: ←	6
Cheung et al. 2020 [39]	China	CC	4–6	295 (162/133)	TGMD-2	LM, OC, FMS	FMS: ↔ LM: ↔ OC: ↔	10
Cliff et al. 2009 [28]	Australia	CS	3–5	46 (25/21)	TGMD-2	LM, OC	LM: → OC: ↔	6
Famelia et al. 2018 [48]	Indonesia	CS	3–6	66 (30/36)	TGMD-3	LM, OC	LM: ↔ OC: ←	7
Freitas et al. 2018 [56]	Portugal	CS	3–6	314 (155/159)	TGMD-2	LM, OC	LM: ↔ OC: ←	8
Hall et al. 2018 [35]	Britain	CS	3–5	166 (91/75)	TGMD-2	LM, OC, FMS	FMS: ↔ LM: ↔ OC: ↔	7
Hall et al. 2019 [36]	Britain	CS	4–6	38 (24/14)	TGMD-2	LM, OC, FMS	FMS: ↔ LM: ↔ OC: ←	8
Henrique et al. 2020 [31]	Brazil	CS	3–5	472 (248/224)	TGMD-2	LM, OC	LM: ↔ OC: ←	7
Jiang et al. 2018 [40]	China	CS	3–6	60 (30/30)	TGMD-2	LM, OC, FMS	FMS: ↔ LM: ↔ OC: ↔	6
Kim et al. 2016 [52]	Korean	CS	5–6	216 (102/114)	TGMD-2	LM, OC	LM: ↔ OC: ←	5
Kit et al. 2017 [63]	United States	CS	3–5	LM: 330 (167/163) OC: 338 (170/168)	TGMD-2	LM, OC	LM: → OC: ←	7
Korbecki et al. 2017 [54]	Poland	CS	6	64 (35/29)	TGMD-2	LM, OC	LM: ↔ OC: ←	5
Kordi et al. 2012 [46]	Iran	QE	4–6	147 (75/72)	TGMD-2	LM, OC	LM: ↔ OC: ↔	7
Lopes et al. 2017 [57]	Portugal	CS	5–6	57 (26/31)	TGMD-2	LM, OC	LM: ↔ OC: ←	8
Mukherjee et al. 2017 [59]	Singapore	CS	6	95 (50/45)	TGMD-2	LM, OC	LM: ↔ OC: ←	6
Nikolić et al. 2016 [44]	Croatia	CS	4–4.5	67 (34/33)	TGMD-1	LM, OC, FMS	FMS: → LM: → OC: ↔	5
Palmer et al. 2020 [64]	USA	QE	3.5–5	54 (27/27)	TGMD-2	LM, OC, FMS	FMS: ↔ LM: ↔ OC: ↔	8
Roscoe et al. 2019 [37]	Britain	CS	3–4	185 (97/81)	TGMD-2	LM, OC, FMS	FMS: ↔ LM: → OC: ↔	6
Saczuk et al. 2021 [55]	Poland	CS	5	441 (255/186)	TGMD-2	LM, OC	LM: → OC: ↔	5
Shi et al. 2020 [41]	China	RCT	5–6	43 (22/21)	TGMD-2	LM, OC, FMS	FMS: ↔ LM: ↔ OC: ↔	9
Soares et al. 2020 [32]	Brazil	CS	3–5	251 (127/124)	TGMD-2	LM, OC, FMS	FMS: ← LM: ↔ OC: ←	7
Tietjens et al. 2018 [45]	Germany	CS	3–6	27 (11/16)	TGMD-3	LM, OC	LM: ↔ OC: ←	6
Tomaz et al. 2019 (1) [60]	South African	CS	3–6	259 (130/129)	TGMD-2	LM, OC, FMS	FMS: ← LM: ↔ OC: ←	7
Tomaz et al. 2019 (2) [61]	South African	CS	3–5	78 (39/39)	TGMD-2	LM, OC, FMS	FMS: ↔ LM: ↔ OC: ←	8
Valentini et al. 2012 [33]	Brazil	CS	3–6	LM: 786 (394/392) OC: 796 (394/402)	TGMD-2-BR	LM, OC	LM: ↔ OC: ←	6
Valentini et al. 2017 [34]	Brazil	CS	3–6	281 (135/146)	TGMD-3-BR	LM, OC	LM: ↔ OC: ←	6
Wang et al. 2020 [42]	China	Cs	3–6	268 (126/142)	TGMD-3	LM, OC, FMS	FMS: ↔ LM: ↔ OC: ↔	8
Webster et al. 2019 [65]	USA	CS	3–4	126 (58/68)	TGMD-3	LM, OC, FMS	FMS: ← LM: ↔ OC: ←	7
Wong & Cheung 2006 [43]	China	CS	3–6	797 (424/373)	TGMD-2	LM, OC	LM: ↔ OC: ←	7

CS: cross-sectional; CC: case–control; QE: quasi-experimental; Cs: cohort study RCT: randomized control trial; TGMD: test of gross motor development; FMS: fundamental movement skills; LM: locomotor skill; OC: object control skill; ←: favors boys; →: favors girls; ↔: no difference.

**Table 2:** The result of publication bias estimation.

Item	Coef.	Std. Err.	t	*p* > |t|	95% Conf. Interval	
FMS	0.8661726	1.340221	0.65	0.529	−2.008316	3.740662
LM	−0.2382333	0.6962142	−0.34	0.734	−1.650221	1.173755
OC	−0.4231878	0.8376626	−0.51	0.616	−2.122046	1.275671

## Data Availability

Not applicable.

## Supplementary Materials

The following supporting information can be downloaded at: https://www.mdpi.com/article/10.3390/ijerph19148318/s1.
