# Supplementary material for: Gender Differences in Fundamental Motor Skills Proficiency in Children Aged 3–6 Years: A Systematic Review and Meta-Analysis
Source: Int J Environ Res Public Health. 2022 Jul 7;19(14):8318. doi: 10.3390/ijerph19148318 (PMC9324170; doi:10.3390/ijerph19148318)
Supplement: Supplementary file 1 [file ijerph-19-08318-s001.zip › Supplementary Materials.pdf]

Search strategy

1.1. Search strategy for PubMed
URL: https://www.ncbi.nlm.nih.gov/pubmed/
Limits: until July 10, 2021

#1 "Child"[MeSH Terms] OR "children"[Title/Abstract] OR "preschoolers"[Title/Abstract] OR "boy"[Title/Abstract] OR "girl"[Title/Abstract]
#2 "Motor Skills"[MeSH Terms] OR "fundamental movement skills"[Title/Abstract] OR "motor development"[Title/Abstract] OR "gross motor"[Title/Abstract]
#3 "TGMD"[Title/Abstract] OR "test of gross motor development"[Title/Abstract]
#4 #1 AND #2 AND #3

1.2. Search strategy for Scopus
URL: https://www.scopus.com
Limits: until July 10, 2021

#1 TITLE-ABS-KEY ("child" OR "children" OR "preschoolers" OR "boy" OR "girl")
#2 TITLE-ABS-KEY ("motor skills" OR "fundamental movement skills" OR "motor development" OR "gross motor")
#3 TITLE-ABS-KEY (TGMD OR "Test of Gross Motor Development")
#4 #1 AND #2 AND #3

1.3. Search strategy for Web of science
URL: https://www.webofscience.com
Limits: until July 10, 2021

#1 TS = (child or children or preschoolers or boy or girl)
#2 TS = (motor skills or fundamental movement skills or motor development or gross motor)
#3 TS = (TGMD or Test of Gross Motor Development)
#4 #1 AND #2 AND #3

Table S1. The Joanna Briggs Institute (JBI) scale quality assessment table

Cross-sectional	Quality assessment								
Authors	Q1	Q2	Q3	Q4	Q5	Q6	Q7	Q8	Scores
Alessandro et al. 2018	U	Y	Y	Y	Y	Y	Y	Y	7
Aponte et al. 1990	U	Y	U	U	Y	Y	Y	Y	5
Aye et al. 2017	Y	Y	Y	Y	Y	U	Y	Y	7
Aye et al. 2018	Y	Y	Y	Y	Y	U	Y	Y	7
Bakhtiar 2014	U	Y	U	Y	Y	Y	Y	Y	6
Behan et al. 2019	U	Y	Y	Y	Y	U	Y	Y	6
Bolger et al. 2017	U	Y	Y	Y	Y	Y	Y	Y	7
Brian et al. 2018	U	Y	U	Y	Y	Y	Y	Y	6

Brian et al. 2019	Y	Y	U	Y	Y	Y	Y	Y	7					
Capio et al. 2021	Y	Y	U	N	Y	Y	Y	Y	6					
Cliff et al. 2009	U	Y	U	Y	Y	Y	Y	Y	6					
Famelia et al. 2018	Y	Y	Y	U	Y	Y	Y	Y	7					
Freitas et al. 2018	Y	Y	Y	Y	Y	Y	Y	Y	8					
Hall et al. 2018	Y	Y	Y	Y	Y	U	Y	Y	7					
Hall et al. 2019	Y	Y	Y	Y	Y	Y	Y	Y	8					
Henrique et al. 2020	U	Y	Y	Y	Y	Y	Y	Y	7					
Jiang et al. 2018	U	Y	Y	Y	Y	U	Y	Y	6					
Kim et al. 2016	U	Y	Y	Y	U	U	Y	Y	5					
Kit et al. 2017	Y	Y	U	Y	Y	Y	Y	Y	7					
Korbecki et al. 2017	U	Y	U	U	Y	Y	Y	Y	5					
Lopes et al. 2017	Y	Y	Y	Y	Y	Y	Y	Y	8					
Mukherjee et al. 2017	U	Y	Y	Y	Y	U	Y	Y	6					
Nikolić et al. 2016	U	Y	Y	Y	U	U	Y	Y	5					
Roscoe et al. 2019	U	Y	Y	Y	Y	U	Y	Y	6					
Saczuk et al. 2021	U	Y	U	Y	Y	U	Y	Y	5					
Soares et al. 2020	U	Y	Y	Y	Y	Y	Y	Y	7					
Tietjens et al. 2018	U	Y	Y	Y	Y	U	Y	Y	6					
Tomaz et al. 2019(1)	U	Y	Y	Y	Y	Y	Y	Y	7					
Tomaz et al. 2019(2)	Y	Y	Y	Y	Y	Y	Y	Y	8					
Valentini et al. 2012	U	Y	Y	N	Y	Y	Y	Y	6					
Valentini et al. 2017	U	Y	Y	N	Y	Y	Y	Y	6					
Webster et al. 2019	U	Y	Y	Y	Y	Y	Y	Y	7					
Wong &Cheung 2006	U	Y	Y	Y	Y	Y	Y	Y	7					
Case–control	Quality assessment													
Cheung et al. 2020	Y	Y	Y	Y	Y	Y	Y	Y	Y	Y	10			
Quasi-experimental	Quality assessment													
Kordi et al. 2012	Y	Y	Y	U	U	Y	Y	Y	Y	7				
Palmer et al. 2020	Y	Y	Y	Y	U	Y	Y	Y	Y	8				
Cohort study	Quality assessment													
Wang et al. 2020	Y	N	Y	Y	Y	Y	Y	U	Y	U	Y	8		
Randomized control trial	Quality assessment													
Shi et al. 2020	Y	U	Y	U	U	U	Y	Y	Y	Y	Y	Y	Y	9
